# *Bifidobacterium adolescentis* Exerts Strain-Specific Effects on Constipation Induced by Loperamide in BALB/c Mice

**DOI:** 10.3390/ijms18020318

**Published:** 2017-02-20

**Authors:** Linlin Wang, Lujun Hu, Qi Xu, Boxing Yin, Dongsheng Fang, Gang Wang, Jianxin Zhao, Hao Zhang, Wei Chen

**Affiliations:** 1 State Key Laboratory of Food Science and Technology, School of Food Science and Technology, Jiangnan University, Wuxi 214122, China; wanglllynn09@163.com (L.W.); 7130112038@vip.jiangnan.edu.cn (L.H.); 6140111107@vip.jiangnan.edu.cn (Q.X.); jxzhao@jiangnan.edu.cn (J.Z.); zhanghao@jiangnan.edu.cn (H.Z.); chenwei66@jiangnan.edu.cn (W.C.); 2 International Joint Research Laboratory for Probiotics, Jiangnan University, Wuxi 214122, China; 3 Kangyuan Dairy Co., Ltd., Yangzhou University, Yangzhou 225004, China; bxyin@yzu.edu.cn (B.Y.); fdsheng@yzu.edu.cn (D.F.); 4 Beijing Innovation Centre of Food Nutrition and Human Health, Beijing Technology and Business University (BTBU), Beijing 100048, China

**Keywords:** *Bifidobacterium adolescentis*, strain-specific, constipation, adhesive, intestinal community structure

## Abstract

Constipation is one of the most common gastrointestinal complaints worldwide. This study was performed to determine whether *Bifidobacterium adolescentis* exerts inter-strain differences in alleviating constipation induced by loperamide in BALB/c mice and to analyze the main reasons for these differences. BALB/c mice underwent gavage with *B. adolescentis* (CCFM 626, 667, and 669) once per day for 17 days. The primary outcome measures included related constipation indicators, and the secondary outcome measures were the basic biological characteristics of the strains, the concentration changes of short-chain fatty acids in feces, and the changes in the fecal flora. *B. adolescentis* CCFM 669 and 667 relieved constipation symptoms by adhering to intestinal epithelial cells, growing quickly in vitro and increasing the concentrations of propionic and butyric acids. The effect of *B. adolescentis* on the gut microbiota in mice with constipation was investigated via 16S rRNA metagenomic analysis. The results revealed that the relative abundance of *Lactobacillus* increased and the amount of *Clostridium* decreased in the *B. adolescentis* CCFM 669 and 667 treatment groups. In conclusion, *B. adolescentis* exhibits strain-specific effects in the alleviation of constipation, mostly due to the strains’ growth rates, adhesive capacity and effects on the gut microbiome and microenvironment.

## 1. Introduction

Constipation is a common health problem and a predisposing factor for many conditions with high death rates. With changes in diet structure and the influence of psychological and social factors in recent years, the incidence of constipation has shown a clear upward trend, which has seriously affected human health and quality of life [1]. Irritant drugs are generally used to promote defecation, but this treatment has side effects [2,3]. Reports have shown that constipation and diarrhea are associated with gut microbes. The relative abundance of pathogens (methanogenic archaea [4] and clostridia [5]) increases in patient with constipation, and the relative abundance of enteropathogenic bacteria (*Salmonella*, *Shigella*, enterotoxigenic *Escherichia coli*, or *Vibrio cholera*) increases in patients with diarrhea [6,7], which disturbs the ecological balance in the intestines [8]. As research into the intestinal micro-ecology continues to develop, it is understandable to attempt treatment of constipation with intestinal micro-ecological therapy.

Increasing fiber intake or using laxatives is commonly recommended to alleviate or treat constipation. The intestinal microbiome is related to normal gastrointestinal (GI) functions such as GI motility, immune modulation, and drug metabolism [9,10,11]. Clinical studies have shown that gut microbiota in constipation differs from that in healthy subjects [12,13] and is mainly manifested in the levels of *Bifidobacterium*, *Lactobacillus* and pathogenic bacteria. Most studies have shown that changes in the intestinal flora in the constipation group mainly involve a decrease in Bifidobacteria and Lactobacilli and an increase in pathogenic bacteria (methanogenic archaea [4] and clostridia [5]). Therefore, supplementation with probiotics has become a new method to treat constipation. Probiotics have been defined as living microbes that, when administered in adequate amounts, such as 10^6^ to 10^9^ colony-forming units (CFU), confer health benefits to the host [14]. Some studies have supported the use of probiotics to prevent or treat constipation [15,16]. Some probiotic strains, either alone—*Bifidobacterium infantis* 36524 or *Lactobacillus plantarum* 299v—or combined—VSL#3 (*Bifidobacterium* (*B. longum*, *B. infantis*, and *B. breve*), *Lactobacillus* (*L. acidophilus*, *L. casei*, *L. bulgaricus* and *L. plantarum*)), and *Streptococcus thermophiles*—have been associated with significant alleviation of constipation [17,18], whereas others have proved ineffective [19,20]. The fundamental reason for the use of probiotics to treat constipation may be that the colonic microflora influences peristalsis of the colon [21]. Therefore, it has been suggested that an imbalance in the colonic microflora plays a role in constipation. Furthermore, Bifidobacteria produce lactic acid and acetic acid, which decrease the pH in the colon. This lower pH enhances peristalsis and decreases the colonic transit time, which is beneficial in the treatment of constipation [16,22]. This latter hypothesis was confirmed by showing a decrease in the colonic transit time in healthy adults who consumed a supplement with *B. animalis* [23].

Numerous probiotic supplementation trials have been carried out in animals and humans to test the efficacy of probiotics against constipation [15,24,25]. It has been shown that Bifidobacteria display inter-species differences in the alleviation of constipation [24]. Bifidobacteria may comprise as much as 25% of the cultivable gut microflora. *B. adolescentis* is recognized as one of the dominant anaerobes in adults and is considered to be beneficial to human health [26]. Therefore, we hypothesized that *B. adolescentis* could alleviate constipation and that inter-strain differences exist in the alleviation of constipation induced by loperamide in BALB/c mice.

Based on this background, the aims of this study were: (1) to determine whether *B. adolescentis* shows inter-strain differences in the alleviation of constipation induced by loperamide in mice; (2) to analyze the main reasons for the inter-strain differences, such as the basic biological characteristics of the strains, the concentration changes of short-chain fatty acids (SCFAs) in feces, and the changes in the fecal flora; and (3) to determine the changes in other indicators of constipation, including some parameters of the enteric nervous system, including motilin (MTL), gastrin (Gas), substance P (SP), endothelin (ET), somatostatin (SS) and vasoactive intestinal peptide (VIP).

## 2. Results

### 2.1. Growth Characteristics of *B. adolescentis* In Vitro

**Figure 1:**
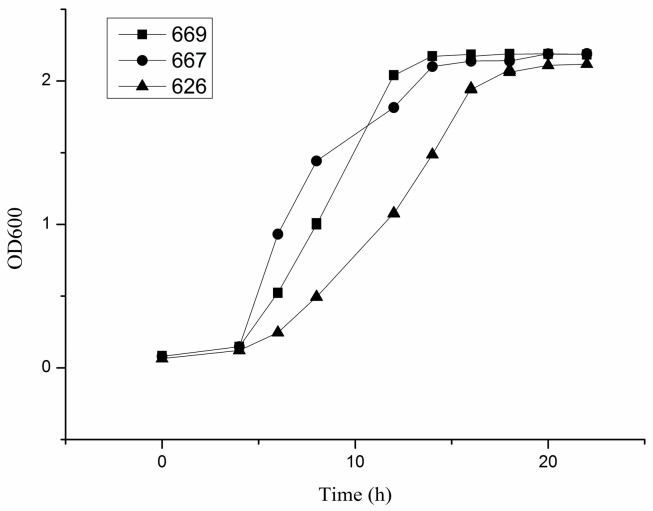
Growth curves of three strains of *Bifidobacterium adolescentis*. The horizontal axis is the time of culturing *B. adolescentis*, and the vertical axis is the absorbance value of the culture solution at 600 nm: 669, the growth curve of *B. adolescentis* 669; 667, the growth curve of *B. adolescentis* 667; and 626, the growth curve of *B. adolescentis* 626. The curves shown are from a single experiment (mean ± SD, *n* = 3).

The growth curve of each strain of *B. adolescentis* cultured under anaerobic conditions at 37 °C in cMRS broth was drawn to determine the growth characteristics of *B. adolescentis* in vitro. The results showed that *B. adolescentis* CCFM 669 and 667 entered the exponential growth phase more quickly than *B. adolescentis* CCFM 626 (Figure 1).

### 2.2. Tolerance Capacity of *B. adolescentis* to Simulated Gastric and Small Intestine Juices

To measure the tolerance capacity of *B. adolescentis* (667, 669 and 626) to gastric acid and bile salts, these three strains were cultured under anaerobic conditions at 37 °C in simulated gastric and intestinal juices. The tolerance capacity of *B. adolescentis* to simulated gastric and intestinal juices is presented in Table 1. Whether in gastric juice or in intestinal juice, the survival rates of the three examined strains showed a decline with the passage of incubation time, but they showed a preferable level of survival and were considered tolerant to gastric and intestinal juices.

### 2.3. Adhesion of Different *B. adolescentis* to HT-29 Cells

HT-29 cells that had been incubated and Bifidobacteria suspension (10^7^ CFU/mL in DMEM) were cultured in six-well tissue culture plates to determine the adherence of *B. adolescentis* to intestinal epithelial cells. The results of three independent experiments performed in triplicate are shown in Table 2. Compared with the positive control *L. plantarum* ST-III, different strains of *B. adolescentis* exhibited different levels of adhesion to HT-29 cells (Table 2): *B. adolescentis* CCFM 669 showed remarkable adhesion, whereas *B. adolescentis* CCFM 626 and 667 showed no adhesion to HT-29 cells.

### 2.4. Effects of *B. adolescentis* on Defecation Status of Mice

**Figure 2:**
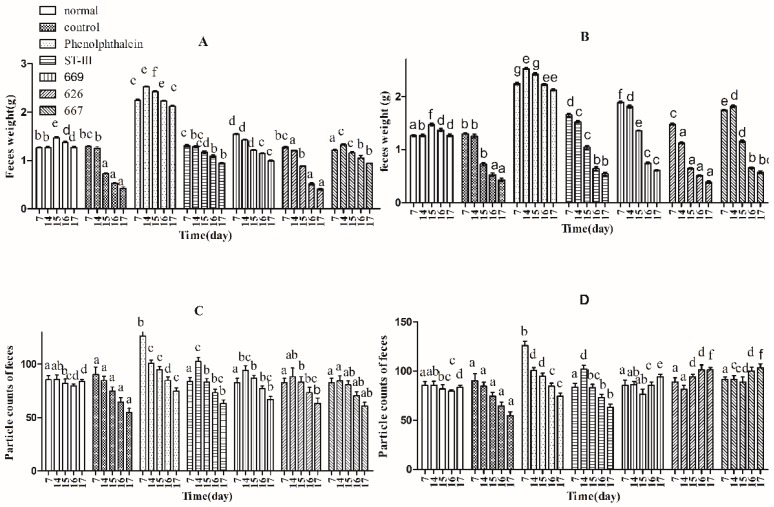

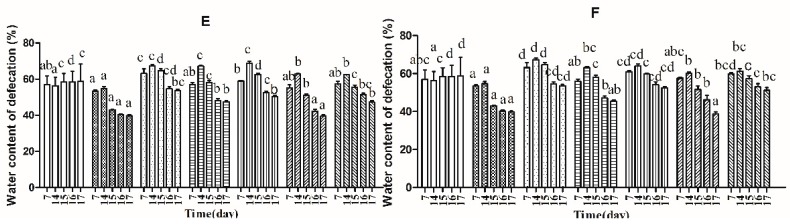
Defecation status of mice during experiments in eight BALB/c mice in each group. Phenolphthalein: 10 mg/kg body weight; 626: *B. adolescentis* CCFM 626; 667: *B. adolescentis* CCFM 667; 669: *B. adolescentis* CCFM 669; ST-III: *Lactobacillus plantarum* ST-III; High dose: 1 × 10^10^ colony-forming units (CFU); Low dose: 1 × 10^8^ CFU. (**A**,**C**,**E**) High-dose groups; and (**B**,**D**,**F**) low-dose groups. Mean values with different letters (a–g) over bars are significantly different (*p* < 0.05) according to one-way analysis of variance and Duncan’s multiple range test.

Three indicators, defecation wet weight, fecal pellet numbers and fecal water content, were used to evaluate defecation status. The results suggest that the defecation weight and fecal pellet numbers in the normal and control groups remained relatively stable before constipation from Day 1 to Day 14, whereas those of the other groups were significantly different by one-way analysis of variance and Duncan’s multiple range test (Figure 2A–D; *p* < 0.05). Furthermore, the water content of defecation in the *B. adolescentis* dose groups, the ST-III group and the phenolphthalein group increased significantly over time (Figure 2E,F; *p* < 0.05), whereas that in the normal and control groups remained stable. Meanwhile, the defecation weight and fecal pellet numbers in the phenolphthalein group were greater than those in the other groups. After constipation was induced by loperamide, the defecation weight, fecal pellet numbers and water content of defecation decreased in all of the treatment groups from Day 15 to Day 17, whereas those in the normal group remained stable (Figure 2A–F; *p* < 0.05). The defecation weight, fecal pellet numbers and water content of defecation increased significantly in the *B. adolescentis* CCFM 667 and 669 dose groups compared to those in the control group (loperamide group). The water content of defecation in the *B. adolescentis* CCFM 626 group was similar to that in the control group, which indicates that *B. adolescentis* CCFM 626 has no effect on constipation. Interestingly, although the particle counts of feces were higher in the low-dose *B. adolescentis* 626 and 667 groups than those in the high-dose groups (Figure 2C,D), the wet weight of total feces was lower in the low-dose *B. adolescentis* 626 and 667 groups than that in the high-dose groups (Figure 2A,B). The feces were dry, hard, and small in the low-dose 626 and 667 groups, indicating that the water content of defecation in the low-dose 626 and 667 groups was lower than that in the high-dose 626 and 667 groups.

### 2.5. Time to the First Black Stool Defecation

**Figure 3:**
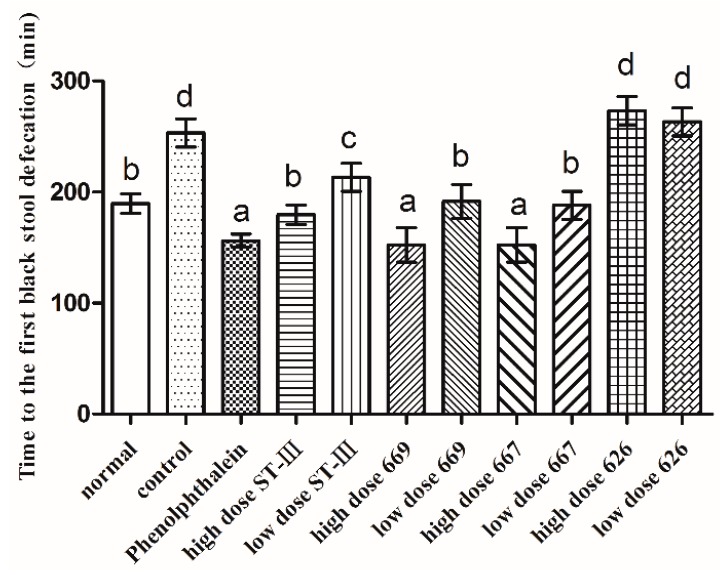
First black stool defecation time of mice after constipation was induced by loperamide in eight BALB/c mice in each group. Phenolphthalein: 10 mg/kg body weight; 626: *B. adolescentis* CCFM 626; 667: *B. adolescentis* CCFM 667; 669: *B. adolescentis* CCFM 669; ST-III: *L. plantarum* ST-III; High dose: 1 × 10^10^ CFU; Low dose: 1 × 10^8^ CFU. Mean values with different letters (a–d) over the bars are significantly different (*p* < 0.05) according to Duncan’s multiple range test.

The time to the first black stool defecation was measured to evaluate the effect of *B. adolescentis* on constipation. The results are shown in Figure 3. The defecation time was significantly different in each group. The time to the first black stool defecation in the normal group was similar to that in the low-dose *B. adolescentis* CCFM 669 and 667 groups and the high-dose ST-III group. Meanwhile, the defecation times in the phenolphthalein group and the high-dose *B. adolescentis* CCFM 669 and 667 groups were the shortest, and those in the control and *B. adolescentis* CCFM 626 groups were the longest, which indicates that *B. adolescentis* CCFM 626 has no effect on constipation.

### 2.6. Gastrointestinal (GI) Transit Rate

**Figure 4:**
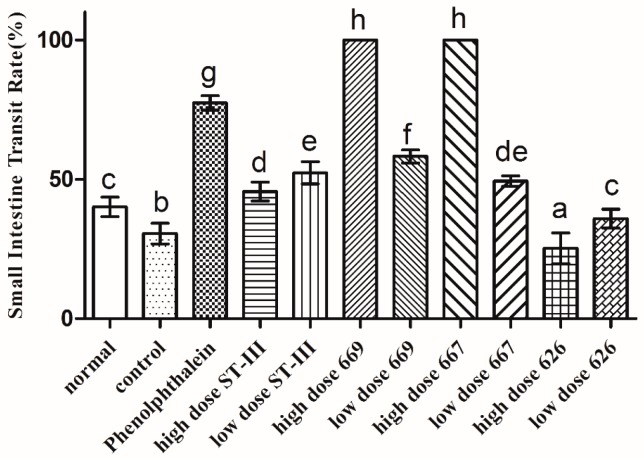
Small intestinal transit rate of mice after constipation was induced by loperamide in eight BALB/c mice in each group. Phenolphthalein: 10 mg/kg body weight; 626: *B. adolescentis* CCFM 626; 667: *B. adolescentis* CCFM 667; 669: *B. adolescentis* CCFM 669; ST-III: *L. plantarum* ST-III; high dose: 1 × 10^10^ CFU; low dose: 1 × 10^8^ CFU. Mean values with different letters (a–h) over the bars are significantly different (*p* < 0.05) according to Duncan’s multiple range test.

The GI transit rate was measured to evaluate the effect of *B. adolescentis* on constipation. As shown in Figure 4, the small intestinal transit rate was significantly different in each group (*p* < 0.05). The transit rate in the control group was decreased compared to that in the normal group, indicating that the constipation model had been established. The two strains of *B. adolescentis* other than *B. adolescentis* CCFM 626 showed significant differences in the small intestinal transit rate. The small intestinal transit rates in the high-dose *B. adolescentis* CCFM 669 and CCFM 667 groups were the highest, that in the phenolphthalein group was the second, and that in the high-dose *B. adolescentis* CCFM 626 group was the lowest. The transit rates in the other groups had improved to different degrees. In conclusion, the three *B. adolescentis* strains showed a strain-specific effect on constipation in mice.

### 2.7. Short-Chain Fatty Acids (SCFAs) in Feces

The concentrations of SCFAs in the feces were determined to assess the effect of *B. adolescentis* on the gut microenvironment in mice with constipation. The results are shown in Table 3. From Day 1 to Day 14, the concentrations of acetic acid, propionic acid, butyric acid and total acid remained stable in the normal and control groups, whereas the other groups showed significant differences (Table 3; *p* < 0.05). The concentrations of propionic acid and butyric acid decreased in the ST-III and *B. adolescentis* CCFM 626 groups, whereas those of the other groups increased significantly. From Day 15 to Day 17, the concentrations of acetic acid, propionic acid, butyric acid and total acid decreased significantly from the first 14 days in the control group, whereas those in the normal group remained stable (Table 3; *p* < 0.05). The concentrations of propionic acid and butyric acid decreased in the *B. adolescentis* CCFM 626 group, whereas those in the other treated groups increased. From Day 1 to Day 17, the concentration of acetic acid increased in all treated groups except for the normal and control groups.

### 2.8. Effect of *B. adolescentis* on Composition of Fecal Microbiota

A data set consisting of 1,660,418 high-quality, classifiable 16S rRNA gene sequences was obtained from 88 fecal samples through MiSeq sequencing analysis. The average sequence read was 13,283 per sample. Representative sequences of all of the sequences were clustered, and a 97% sequence similarity cut-off was used. The number of operational taxonomic units (OTUs) per sample ranged from 791 to 6386.

**Figure 5:**
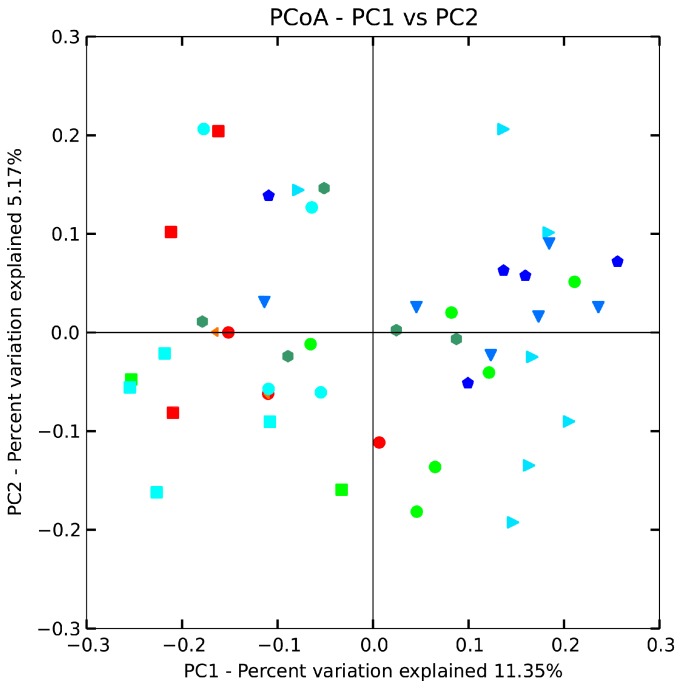
Principal coordinates analysis (PCoA) plots based on unweighted UniFrac metric. Each colored symbol represents the composition of fecal microbiota of one mouse. Symbol: 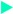, control; 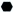, normal; 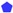, phenolphthalein; 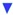, ST-III; 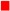, high-dose 667; 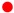, low-dose 667; 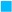, high-dose 669; 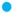, low-dose 669; 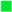, high-dose 626; 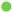, low-dose 626.

The beta diversity of gut microbiota in mice treated with *B. adolescentis* (CCFM 626,669 and 667) at the genus level was revealed by using an unweighted uniFrac matrix (Figure 5). However, the difference between the control, low-dose 626 and phenolphthalein groups was smaller, indicating that the taxa with the highest relative abundance were more similar between these three groups than the taxa that accounted for a smaller proportion of the community. The data points shifted from the right of the score plot to the left in two doses of 667 and 669 groups, indicating that *B. adolescentis* 669 and 667 changed the gut microbiota structure of mice. To test the hypothesis that the composition changed significantly, we performed one-way nonparametric multivariate analysis of variance with the total microbiota composition data. The results showed significant differences between the *B. adolescentis* CCFM 669, CCFM 667 and control groups, whereas the differences between the 626 group and control group were not statistically significant. In other words, *B. adolescentis* CCFM 669 and CCFM 667 significantly changed the gut microbiota structure of mice (Table 4). Interestingly, no significant differences were observed between the low-dose 669 group and the normal group. This result revealed that the gut microbiota of constipated mice treated with low-dose 669 recovered to normal status.

**Figure 6:**
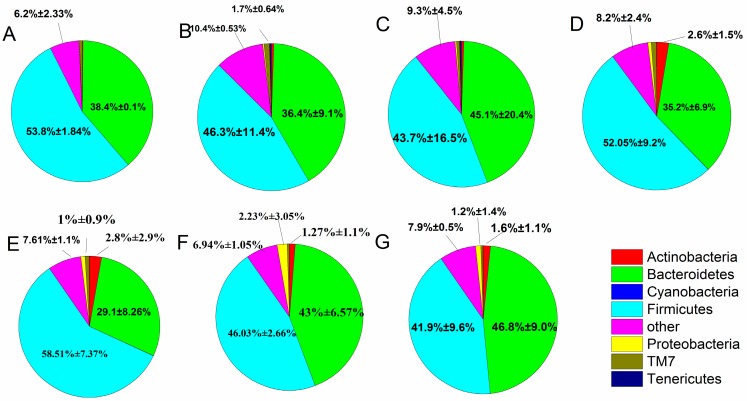
Relative abundance of main phyla in the different groups after constipation: (**A**) normal; (**B**) control; (**C**) phenolphthalein; (**D**) ST-III; (**E**) *B. adolescentis* CCFM 669; (**F**) *B. adolescentis* CCFM 667; and (**G**) *B. adolescentis* CCFM 626.

When individual OTUs were used to combine the phylum level (Figure 6), the intestinal flora was mostly dominated by Firmicutes and Bacteroidetes, which accounted for 88.04%, and Proteobacteria and Actinobacteria (3.89%). The fecal microbiota changed between the treatment, control and normal groups, as described in the following subsections. After constipation, the relative abundance of Firmicutes in the control group decreased compared with the normal group (from 53.8% to 46.3%), whereas the *B. adolescentis* CCFM 669 and ST-III groups showed an increase in the relative abundance of Firmicutes (from 46.3% to 58.51% and 52.05%) and a significant decrease in others (from 10.4% to 7.61% and 8.2%) (*p* < 0.05) compared with the control group by one-way analysis of variance. The relative abundance of Firmicutes and Bacteroidetes was similar in the *B. adolescentis* CCFM 667 group (46.03% vs. 43%) and the phenolphthalein group (43.7% vs. 45.1%). The *B. adolescentis* CCFM 626 group showed a decrease in Firmicutes and an increase in Bacteroidetes compared with the normal group.

**Figure 7:**
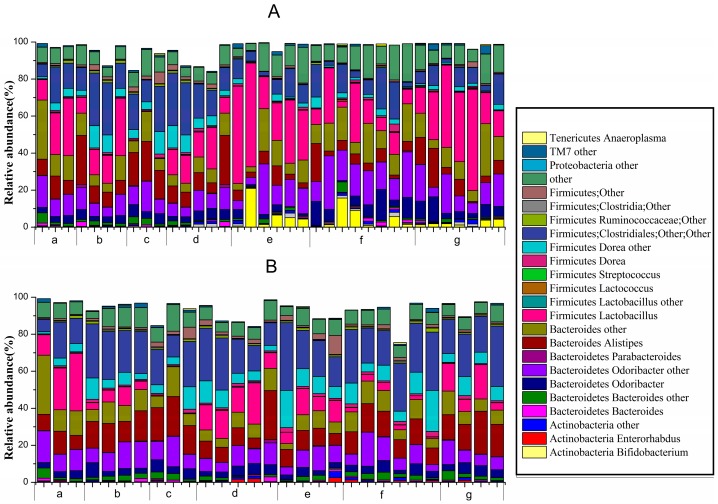
Relative abundance of main genera >0.1% at d1422-0067/18/02/0317/s2ifferent periods in different groups: (**A**) before constipation (Days 1 to 14); and (**B**) during constipation (Days 15 to 17) ((**a**) normal group; (**b**) control group; (**c**) phenolphthalein group; (**d**) ST-III group; (**e**) *B. adolescentis* CCFM 669 group; (**f**) *B. adolescentis* CCFM 626 group; and (**g**) *B. adolescentis* CCFM 667 group).

At the genus level (Figure 7), unknown genera belonging to Actinobacteria (*Bifidobacterium*, *Enterorhabdus* and others), Bacteroidetes (*Bacteroides*, *Odoribacter*, *Parabacteroides*, *Alistipes* and others), Firmicutes (*Lactobacillus*, *Lactococcus*, *Dorea*, *Clostridiales*, *Ruminococcaceae* and others) and Proteobacteria, TM7 and Tenericutes (*Anaeroplasma*) all comprised >0.1% of the microbiota.

**Figure 8:**
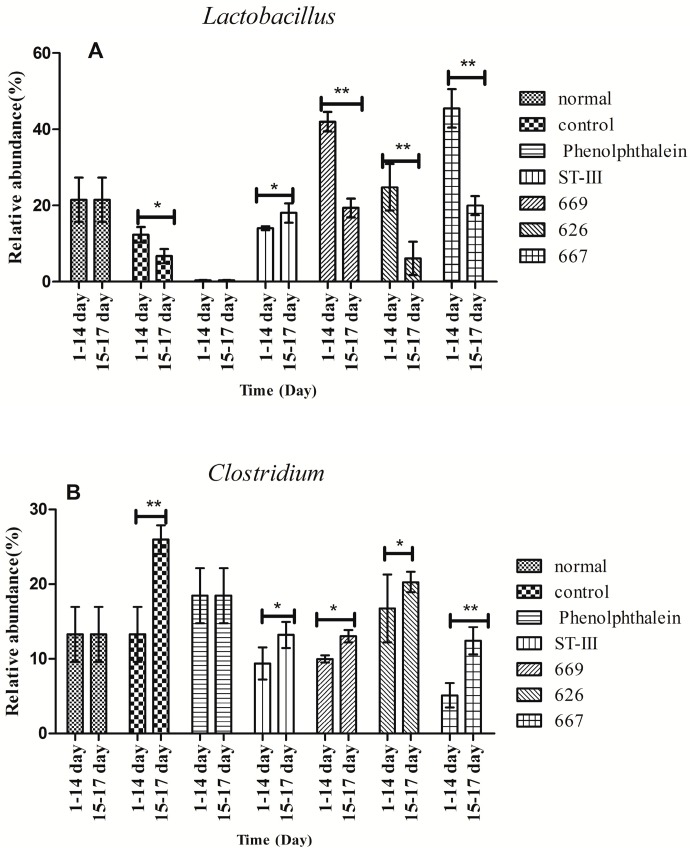
Changes in abundance of selected genera at different periods. * *p* < 0.05; ** *p* < 0.01 according to one-way analysis of variance and Duncan’s multiple range test.

Before constipation (Day 1 to Day 14), the fecal flora structure changed tremendously during the two-week administration of *B. adolescentis* as suggested by the results of the one-way analysis of variance and Duncan’s multiple range test (Figure 7A and Figure 8). As expected, the relative abundance of *Bifidobacterium* increased remarkably from 0.24% to an average of 4.62% (shown in yellow in the histogram) (*p* < 0.01). Meanwhile, the level of *Lactobacillus* was significantly increased from 21.54% to an average ratio of 37.5% (shown in fuchsia in the histogram, Figure 8) (*p* < 0.05) during the *B. adolescentis* treatment periods. In addition, the levels of many other bacteria, such as *Alistipes*, *Dorea* and *Clostridium*, decreased after two weeks of *B. adolescentis* treatment. 

After loperamide-induced constipation (Days 15 to 17), the fecal flora structure changed slightly compared with that from the first 14 days (Figure 7B). The levels of *Lactobacillus* (Figure 7) and *Bifidobacterium* decreased in the *B. adolescentis* treatment groups but were still higher than that in the control group. In addition, *Alistipes* and *Dorea* tended to revert to their original levels before *B. adolescentis* treatment, as indicated by the cluster results between the data points of Figure 6A,E–G, and Figure 6B,E–G. The relative abundance of *Clostridium* showed a significantly decrease compared to the normal and phenolphthalein groups as suggested by the results of the one-way analysis of variance and Duncan’s multiple range test (Figure 8).

### 2.9. Characterization of Serum Parameters of Experimental Mice

The effects of *B. adolescentis* on constipation were further evaluated by measurement of serum parameters in the experimental mice, including MTL, Gas, SP, ET-1, SS and VIP. As shown in Table 5, no statistical differences were seen in these six indices between the *B. adolescentis* CCFM 626 group and the control group. In the treatment groups, the levels of MTL, Gas and SP were significantly increased, whereas the levels of ET-1, SS and VIP were significantly decreased (*p* < 0.05).

## 3. Discussion

Constipation is a common functional GI disorder whose main clinical symptoms include difficulty with defecation, reduced defecation frequency, dry and hard stools and a prolonged GI emptying time [27]. Patients with constipation have severe disturbances of intestinal flora and a large number of pathogen-produced nitrite amines, phenols, ammonia, azobenzene and carcinogenic substances. Direct contact with some of these harmful substances can cause inflammation of intestinal mucosal or even colon cancer. These toxins cannot be discharged in time and can be absorbed into the blood to induce breast cancer [28]. Disruption of the intestinal microflora balance may modify the intestinal barrier function and influence health. A micro-ecological view showed that a sufficient quantity of Bifidobacteria in the gut can ferment oligosaccharides and produce acetic acid and lactic acid to promote intestinal peristalsis, excretion of feces, and alleviation of constipation [29]. The fecal wet weight, the fecal water content, the time to the first black stool defecation and the rate of intestinal charcoal propulsion are important indices with which to evaluate the function of the GI tract.

The purpose of this study was to determine whether *B. adolescentis* exerts a strain-specific effect on constipation and the causes of these differences. Our study revealed that the three strains of *B. adolescentis* exerted a strain-specific effect on constipation caused by loperamide, and these differences were mainly caused by the fundamental properties of the strains and their effects on the intestinal flora and intestinal microenvironment.

A mouse constipation model was established by administration of loperamide, which is an agonist of μ-opioid receptors that prevents the release of acetylcholine and prostaglandin, resulting in inhibition of intestinal peristalsis and prolonged retention of the intestinal contents. Therefore, loperamide-induced constipation is considered a model of spastic constipation [30]. In this study, the loperamide (control) group showed evident symptoms of constipation, including significant decreases in fecal wet weight, fecal water content, small intestinal transit rate, and time to the first black stool defecation in comparison to the normal group. These symptoms were relieved in the *B. adolescentis* CCFM 669 and 667 treatment groups, but not in the *B. adolescentis* CCFM 626 treatment group. This finding indicates that *B. adolescentis* has a strain-specific effect on constipation.

There are three modes of small bowel movement: tension contraction, segmentation movement and peristalsis. The movement of intestinal contents is mainly promoted by peristalsis, which is detected by measuring the small intestine propulsion rate. A faster small intestine propulsion rate is conducive to the discharge of feces; otherwise, constipation is likely [31]. Our study revealed that *B. adolescentis* CCFM 667 and 669 relieve constipation by improving the small intestine propulsion rate. Although the high dose of *B. adolescentis* CCFM 626 did not alleviate constipation, the low dose could, which showed that a larger dose did not lead to a better effect; therefore, screening for the optimum dose was the next task. Previous studies have confirmed the efficacy of treatment with *B. lactis* DN-173010, *B. longum* 46 (DSM 14583), *B. longum* 2C (DSM 14579) and *B. lactis* HN019 (DR10TM) on the frequency of defecation and stool consistency [32,33]. All of these studies indicated that Bifidobacteria could relieve constipation by improving the fecal status and the rate of intestinal propulsion.

The whole intestinal transit time is reflected by the time to the first black stool defecation, which is the sum of the transit times of the small and large intestine. The main cause of constipation is the long retention time of feces in the intestinal tract and excessive absorption of water. The time to the first black stool defecation is an important indicator to judge both the effect of treatment and prognosis. A shorter time to the first black stool defecation indicates a better effect; otherwise, the effect will be worse. Our study showed that the time to the first black stool defecation was the shortest in the group that received a high dose of *B. adolescentis* CCFM 667 or 669 or phenolphthalein and the longest in the control and *B. adolescentis* CCFM 626 groups. Thus, CCFM 667 and 669 could relieve constipation, whereas CCFM 626 could not. Published data hold that supplementation of *Bifidobacterium* is associated with a slower whole intestinal transit time in the human gut [34], a finding that was confirmed by Favretto [35].

The water content of feces and the small intestinal transit rate were significantly greater in the low-dose *B. adolescentis* CCFM 669 group than those in the low-dose *B. adolescentis* CCFM 667 group, and the time to the first black stool defecation was similar to that in the *B. adolescentis* CCFM 667 group. These results revealed that *B. adolescentis* CCFM 667 relieves constipation by improving the colonic transit time and that *B. adolescentis* CCFM 669 relieves constipation by promoting small intestinal peristalsis. Furthermore, *B. adolescentis* CCFM 667 did not have adhesion ability, so it passed into the large intestine with chyme. It would quickly produce SCFAs from carbohydrates that could not be absorbed by the small intestine and would stimulate colon peristalsis, ultimately accelerating colonic transit time. The action of *B. adolescentis* CCFM 669 was completely contrary to that of *B. adolescentis* CCFM 667. It partly colonized in the small intestine, promoted intestinal peristalsis and improved the small intestinal transit rate. The remainder passed into the large intestine with chyme and played the same role as *B. adolescentis* CCFM 667.

The changes in the intestinal flora in mice were mainly detected by the changes in the bacteria in their feces. How do mouse intestinal flora change before and after constipation, and after supplementation with *B. adolescentis*? The results clearly showed that the composition of the gut microbiota community both before and after intervention was dominated by two phyla, Bacteroidetes (which includes *Bacteroides* and *Odoribacter*) and Firmicutes (which includes *Clostridium*, *Streptococcus*, *Lactobacillus* and *Dorea*), whereas Actinobacteria (mainly *Bifidobacterium*), Proteobacteria and Tenericutes played minor roles. In general, at the phylum level, the ratio of Firmicutes to Bacteroidetes increased and the abundance of other phyla decreased in the *B. adolescentis* treatment groups. Similarly, at the genus level, *B. adolescentis* did not increase the abundance of *Bifidobacterium* in Actinobacteria but did increase the relative abundance of *Lactobacillus* in Firmicutes and reduced the abundance of *Clostridium*. Some species of the *Clostridium* genera are associated with the emergence of intestinal complications such as colitis [36], necrotizing enterocolitis and gastroenteritis [37]. Therefore, the reduction of harmful *Clostridium* after *B. adolescentis* ingestion indicates a potential benefit for gut health. The intestinal flora comprises a complex and dynamic bacterial community that plays an important role in human health [38]. Previous studies described the changes in the gut microbiota of patients with constipation, which are characterized by a relative decrease in the *Bifidobacterium* and *Lactobacillus* species and an increase in potentially pathogenic microorganisms [39]. Previous studies reported no significant differences in the microbial profiles of patients with constipation and healthy subjects [4]. The conflicting results might also relate to differences in the definition of constipation in the various studies. We measured objective indices such as the whole intestinal transit time rather than more subjective indices. For example, Khalif et al. [39] observed little difference in the gut bacteria between healthy subjects and those with constipation, but only constipated subjects with a severely prolonged transit time were considered in the comparison. It is possible that symptom-based diagnosis such as that with a subjective index may not be sufficient to differentiate gut microbial differences between subjects with and without constipation. In brief, altering the composition of the gut microbiota community in patients with constipation to resemble that in normal subjects may decrease constipation-associated changes in GI function.

Microbes do not simply remain within the gut; they must be metabolically active to survive in that environment. Hence, *B. adolescentis* would have an influence not only on the composition and numbers of various microbes, but also on their fermentation products, such as SCFAs. SCFAs are the final product of microbial fermentation in the mammalian colon, in which they represent the major organic anions. The ability of the bacteria to produce SCFAs is influenced by the number of bacteria, the pH and the substrate. The total amount and proportion of SCFAs produced by different substrates differ [40]. SCFAs are involved in important physiological metabolic processes in vivo [41]. Thus, measuring the content of SCFAs in the intestine has become the main method to detect differences between strains in the relief of constipation. Our study showed that *B. adolescentis* increased the concentration of propionic acid and butyric acid to relieve constipation. Propionate is primarily used in the liver and has been suggested as a potential modulator of cholesterol synthesis and a precursor in lipo-neogenesis, which may influence body weight [42]. Butyrate is the preferred energy source for colonocytes and thus is extensively metabolized by the colon. Butyrate and other SCFAs have also been shown to provide protection against colon cancer [43]. Compared with other similar investigations, some have observed significantly higher levels of isobutyrate in samples from subjects with constipation than in those from healthy subjects [4], and others reported increases in butyrate levels [44]. These phenomena might be related to diet because SCFAs originate from the degradation of polysaccharides, which increases when the system operates with longer retention times [45]. Our study incorporated dietary and gut transit time, and the results are more reliable. Further studies are also needed to provide direct evidence for increased propionate and butyrate acid and to examine the possible role of propionate and butyrate in the pathogenesis of constipation. As to the molecular mechanism of the beneficial effects of SCFAs, butyrate was reported to be a physiological regulator of major pathways of colonic epithelial cell maturation including cell cycle arrest, lineage-specific differentiation, and apoptosis [46]. In addition, increased expression of genes that favor apoptosis (Bax, and Bak) and a reduced expression of counter-players that prevent apoptosis (Bcl-2, and Bcl-XL) were all reported to be the molecular mechanisms of SCFAs that mediate colorectal cancer [47]. Compared to the in-depth investigation of the molecular mechanism of SCFAs intervention in colorectal cancer, few studies have reported molecular evidence of the mediation of SCFAs in constipation. It is believed that SCFAs may stimulate water and electrolyte absorption, potentiate the proliferation of epithelial cells, influence GI motility, increase mesenteric blood flow and exert other physiological effects [41]. However, the molecular mechanism still needs to be investigated.

GI hormones such as MTL, Gas, SP, ET, SS, and VIP play important roles in the regulation of GI motility [48] and have been implicated to different extents in normal and pathophysiological situations. MTL, Gas and SP are excitatory peptide neurotransmitters, whereas ET, SS and VIP are inhibitory peptide neurotransmitters. In this study, the control group showed evident constipation symptoms in relation to neurotransmitters, including significantly decreased levels of MTL, Gas and SP and increased levels of ET, SS and VIP in comparison to the normal group and the *B. adolescentis* CCFM 669 and 667 groups, whereas no statistical differences were seen between the *B. adolescentis* CCFM 626 group and the control group. *B. adolescentis* not only improved the symptoms of constipation and changed the mice’s intestinal flora and microenvironment, but also affected the GI neurotransmitters related to constipation. MTL influences the transport of water and electrolytes, promotes gastric contractions and small intestine segmental movement, accelerates intestinal transfer time and increases colon movement. Gas stimulates the secretion of gastric acid and pepsinogen and promotes the growth of digestive tract mucosa, the contraction of the GI smooth muscle and the relaxation of the pyloric sphincter. SP adjust the contraction of the GI tract, intestinal motility and gastric acid secretion. Therefore, the promotion of the serum levels of MTL, Gas and SP accelerates intestinal peristalsis and the transport of contents. In our study, the levels of MTL, Gas and SP in the control group were significantly decreased compared with those in the normal and *B. adolescentis* treatment groups. This phenomenon confirmed that the decreases in MTL, Gas and SP might be amongst the causes of constipation. These results are consistent with those of several other reports [49,50,51].

ET, as a multifunctional peptide, can exert important effects on numerous aspects of cardiovascular, neuroendocrine and gastrointestinal function [52]. Meanwhile, it plays an important role in the stability of vascular tension and maintains the basic cardiovascular system. SS inhibits the release of GI hormones, such as MTL and Gas, and the secretion of gastric acid, trypsin and amylase. Vecht [53] revealed that SS could slow the small intestinal transit time significantly, whether during eating or fasting. VIP is a polypeptide composed of 28 amino acids whose function is to relax the GI tract and GI sphincter, and it significantly promotes the colon cancer induced by carcinogens in mice. Fasth et al. [54] held that VIP is an important factor in the production of descending inhibition, resulting in slow transmission. Studies have found that the levels of ET, SS and VIP in control groups were higher than those in normal and *B. adolescentis* treatment groups, which means that *B. adolescentis* influenced the level of GI hormones, an index that could reflect the status of constipation.

## 4. Materials and Methods

### 4.1. Chemicals and Reagents

Kits used to measure the levels of MTL, Gas, SP, ET, SS, and VIP were purchased from Wen LE Bioengineering Institute (Shanghai, China). MRS broth was purchased from Qingdao Hopebio Company (Qingdao, China). Dulbecco’s minimum essential medium–high glucose (DMEM), fetal bovine serum, penicillin, and streptomycin were obtained from HyClone (Logan, UT, USA).

Loperamide hydrochloride, (2 mg per capsule) was purchased from Wuxi big drugstore (Xi’an Janssen Pharmaceutical Ltd., Xi’an, China). Loperamide was dissolved in distilled water to reach its final concentration of 1 mg/mL. Phenolphthalein (0.1 g, 100 tablets) was dissolved in distilled water to a final concentration of 7 mg/mL.

For activated carbon meal solution, gum arabic 100 g and water 800 mL were boiled until the solution was transparent. Activated carbon 50 g was then added and boiled three times. After the solution was cool, it was diluted with water to 1000 mL, stored at 4 °C, and mixed before use. All other chemicals and reagents used in this study were of analytical grade. The *Bifidobacterium* species used in this study were obtained from different samples in different regions and kept in the culture collection of food microorganisms of Jiangnan University (Wuxi, China).

### 4.2. Bacterial Strain and Culture Conditions

The *Bifidobacterium* species used in this study, including *B. adolescentis* CCFM 626, 667 and 669, were stored at −80 °C in 30% (*v*/*v*) glycerol broth and then cultured under anaerobic conditions for 24 to 48 h at 37 °C in modified MRS (cMRS) broth supplemented with 0.05% *w*/*v* l-cysteine-HCl (Merck) [55]. To prepare active cultures for all experiments, all strains were consecutively reactivated in an anaerobic atmosphere at least three times using 3% (*v*/*v*) inoculum in cMRS broth at 37 °C for 24 to 48 h before use.

To use these strains in the animal experiments, the bacterial culture was centrifuged at 3000× *g* for 10 min, washed twice with sterile saline solution, and centrifuged again at 3000× *g* for 10 min to obtain the bacteria. The bacteria were lyophilized using skimmed milk as a protectant and stored at −20 °C. The viability of the freeze-dried cells was measured by colony counting before the animal experiments. The bacterial suspensions were prepared daily by diluting 4 × 10^10^ CFU of freeze-dried *B. adolescentis* in sterile saline solution before the animal experiments.

### 4.3. Growth Curve of *B. adolescentis*

An early stationary phase culture of each *B. adolescentis* strain (about 10^7^ CFU/mL) was inoculated into fresh cMRS medium. The cultures were grown in an anaerobic atmosphere at 37 °C, and OD600 values were measured every 2 h. The growth curve of each *B. adolescentis* culture was made with the incubation time as the abscissa and the corresponding absorbance values as the ordinate.

### 4.4. Tolerance Capacity of B. adolescentis to Simulated Gastric and Small Intestine Juices

Simulated gastric juices were prepared with 3 g/L pepsin (1:10,000, Sigma, St. Louis, MO, USA) in phosphate-buffered saline solution (PBS; pH 3.0) and filtered with a 0.22-μm filter before use. Simulated small intestinal juices were prepared with 1 g/L trypsin (1:250, Sigma, St. Louis, MO, USA) in PBS (pH 8.0) and filtered with a 0.22-μm filter before use. Washed cell suspensions (1 mL) of *B. adolescentis* were mixed with 9 mL of the simulated gastric juice and cultured at 37 °C in anaerobic conditions. The samples were periodically withdrawn at 0, 1, 2 and 3 h, and a 1-mL sample was then transferred into 9 mL of sterile saline solution by serial dilutions. A 0.1-mL portion of the suspension was cultured anaerobically on a cMRS agar plate at 37 °C for 48 h. Strains sampled at 0 h were used as a control. CFUs were counted, and the survival rate in gastric juices was determined [56]. Washed cell suspensions (1 mL) sampled at 3 h in gastric juice were then inoculated into 9 mL of sterile small intestine juice and cultured in anaerobic conditions at 37 °C. A sample volume of 1 mL was taken at 2, 4 and 8 h, transferred into 9 mL of sterile saline solution and mixed. The survival rate in the intestinal juice was then detected with the same manipulation.

$$\mathrm{survival}\;\mathrm{rate}(\%)=\frac{\log cfuN_{1}}{\log cfuN_{0}}\times 100\%$$

where *N*_1_ = total viable count of *B. adolescentis* strains after treatment with simulated GI juices and *N*_0_ = total viable count of *B. adolescentis* strains before treatment.

### 4.5. Determination of Adhesion Properties of Bifidobacterium spp. to HT-29 Cells In Vitro

The adherence of *B. adolescentis* to intestinal epithelial cells of the HT-29 cell line was examined as described by Coconnier et al. [57]. In brief, HT-29 cells were purchased from the cell bank of the type culture collection of the Chinese Academy of Sciences (Shanghai, China). The cells were grown in DMEM containing 10% heat-inactivated (30 min at 56 °C) fetal bovine serum, 100 U/mL penicillin and 100 U/mL streptomycin at 37 °C in a humidified atmosphere of 5% CO_2_ and 95% air. For the adherence assays, the HT-29 cells were cultured in six-well tissue culture plates without antibiotics. Approximately 10 h after incubation, the HT-29 cells were carefully collected and washed three times with sterile PBS (pH 7.8), and 1 mL fresh DMEM and 1 mL of Bifidobacteria suspension (10^7^ CFU/mL in DMEM) were then added. The inoculated cultures were incubated for 3 h at 37 °C in 5% CO_2_ and 95% air. The treated cells were washed three times with sterile PBS (pH 7.8), fixed in methanol, gram-stained and observed microscopically. The strain *L. plantarum* ST-III was used as a control.

### 4.6. Animal Experiments

#### 4.6.1. Animals

Seven-week-old male BALB/c mice (initial body weight, 18 to 21 g) were purchased from Jiangsu Laboratory Animal Centre (Suzhou China) and used in all of the experiments; they were fed under standard conditions at a room temperature of 25 ± 2 °C and humidity of 50% ± 5% with a 12-h light–dark cycle. All protocols for this study were approved by the Ethics Committee of Jiangnan University, China (JN. No20150326-0110-21). The procedures were carried out in accordance with the European Community guidelines (Directive 2010/63/EU) for the care and use of experimental animals.

#### 4.6.2. Induction of Constipation and Experimental Design

After adaptation to the environment for 7 days, mice were fed with standard diet (Nanjing Qinglong animal farm, Nanjing, China), and 88 mice were used to investigate the strain-specific preventive effects of *B. adolescentis* in constipation. The mice were randomly divided with eight mice in each of 11 groups: the normal group (saline solution or distilled water), the constipation control group (loperamide 10 mg/kg body weight), two groups with different doses of *B. adolescentis* CCFM 626, 667 or 669 (i.e., 1 × 10^10^ CFU or 1 × 10^8^ CFU *B. adolescentis* 626, 667 or 669; for a total of six groups), a positive drug control group (phenolphthalein 70 mg/kg body weight [58]) and two positive control groups (*L. plantarum* ST-III at either a high dose of 1 × 10^10^ CFU or a low dose of 1 × 10^8^ CFU).

The experiment was designed as follows. All animals were fasted overnight (approximately 18 h) before the first experiment (water was not restricted). The control groups were given normal saline solution once per day via gavage for 17 days. The high-dose *B. adolescentis* CCFM 626, CCFM 667 and CCFM 669 and *L. plantarum* ST-III groups and the low-dose *B. adolescentis* CCFM 626, CCFM 667 and CCFM 669 and *L. plantarum* ST-III groups received 0.25 mL of 4 × 10^10^ CFU/mL or 4 × 10^8^ CFU/mL bacterial suspension, respectively, in the same manner as the treatment groups for 14 days, and the positive drug control group was treated with a 70 mg/kg body weight dose of phenolphthalein once per day for 14 days. Phenolphthalein is a laxative drug that acts to stimulate intestinal peristalsis. It is typically prescribed for the relief of constipation and for the management of neurogenic bowel dysfunction [59]. The control and treatment groups were treated with loperamide (10 mg/kg body weight; 0.25 mL) via gavage from Day 15 to 17 to induce constipation [60]. Fecal water content, the time to the first black stool defecation, GI transit (the rate at which carbon powder is propelled in the small intestine), SCFAs and gut microbiota in feces were measured. The excreted feces of the individual mice were collected in a tube and their wet weight was measured immediately after excretion every day during the experimental period. The feces were then thoroughly dried and weighed, and the water content of the fecal pellet was calculated as the difference between its wet and dry weights. The specific arrangements of the experiment are shown in Table 6.

#### 4.6.3. Measurement of Defecation Status of Mice

During the 17-day experimental period, each mouse was moved into a clean, empty cage every day for 3 h, during which stool samples were collected, counted, and weighed. The water content was calculated as the difference between the wet and dry weights of the stool as described previously [61]. This measurement was performed to determine the effect of *B. adolescentis* in improving intestinal motility and feces status.

#### 4.6.4. Determination of the Time to the First Black Stool Defecation

The mice were fasted overnight with water provided; after 18 h, the mice were treated with loperamide (10 mg/kg body weight, 0.25 mL) or normal saline solution (normal group), and 1 h later, all mice were treated with activated carbon meal by the same method. The animals were then immediately moved into clean, empty individual cages and allowed food and water ad libitum. The length of time from the administration of activated carbon meal to the appearance of darkened feces was recorded.

#### 4.6.5. Determination of GI Transit

The small intestinal transit time was measured with the method of Nagakura et al. [62], with minor modification. Mice were fasted overnight (approximately 18 h, with water provided) from Day 17 at 6:00 p.m. After 16 h, 0.25 mL of activated carbon meal was administered to each mouse via gavage. One hour later, the mice were killed with light ether anesthesia, and their blood was collected in tubes for almost 2 h and centrifuged at 3000× *g* for 15 min to obtain serum. The abdomen was opened, and the entire small intestine starting from the pylorus to the cecum was carefully removed and placed on blotting paper. The distance over which the activated carbon had travelled and the total length of the small intestine were measured. The GI transit of each mouse was calculated as the percentage of the distance travelled by the activated carbon meal relative to the total length of the small intestine. 

#### 4.6.6. Determination of SCFAs in Feces

The concentrations of SCFAs were determined according to Mao et al. by gas chromatography mass spectrometry (GCMS-QP2010 Ultra system, Shimadzu Corporation, Kyoto, Japan) [63], which was equipped with a Rtx-Wax column (30 m × 0.25 μm × 0.25 μm). The carrier gas was helium, the flow rate was 2 mL/min, the split ratio was 10:1 and the volume of sampling was 1 μL. The injection temperature was 240 °C, and the GC temperature program was as follows: the initial temperature of 100 °C was increased to 140 °C at the rate of 7.5 °C/min, and then was increased by 60 °C/min to 200 °C, which was maintained for 3 min. The ionization temperature was 220 °C. Mass scanning was performed in full scan mode. A standard curve was generated by the external standard method, and the concentrations of SCFAs were calculated according to the standard curve in units of μmol/g sample. Fecal samples separately collected from each mouse, were subjected to soaking, acidification, and extraction using saturated NaCl solution, sulfuric acid (10%), and diethyl ether, respectively, and the concentration of total SCFAs consisting of acetic acid, propionic acid and butyric acid was calculated.

#### 4.6.7. MiSeq Genome Sequencing Analysis of Community Structures

Metagenomic DNA from the fecal samples was obtained using a FastDNA Spin Kit for Soil (MP Biomedical, catalog No. 6560-200) following the manufacturer’s instructions. The V4 region of the 16S rRNA was amplified from microbial genome DNA (forward primer, 5′-AYTGGGYDTAAAGNG-3′; reverse primer, 5′-TACNVGGGTATCTAATCC-3′) as described previously by polymerase chain reaction [64]. The products were excised from a 1.5% agarose gel, purified by Gene Clean Turbo (MP Biomedical, Beijing, China) and quantified with a Quant-iT PicoGreen dsDNA Assay Kit (Life Technologies, Carlsbad, CA, USA) according to the manufacturer’s instructions. Libraries were prepared using TruSeq DNA LT Sample Preparation Kit (Illumina, San Diego, CA, USA) and sequenced for 500 + 7 cycles on an Illumina MiSeq using the MiSeq Reagent Kit. The read length is 2 × 250 bp.

After sequencing, 16S rRNA reads were analyzed using the QIIME pipeline. [65] The raw sequences were screened. The short lengths (<200 bp) were then removed, and the pair-end reads that overlapped longer than 10 bp and without any mismatch were assembled according to their overlap sequence. For the high quality sequences obtained by assembling, the sequences with similarity greater than 97% were defined as an OTU by QIIME software (http://qiime.sourceforge.net/), and finally, representative sequences of OTUs were used to identify a species. The beta diversity of microbial communities was investigated by visual assessment using principle coordinate analysis plots and by an analysis of similarity calculated based on weighted UniFrac distances (QIIME) according to one-way nonparametric multivariate analysis of variance.

#### 4.6.8. Determination of MTL, Gas, ET, SS, SP and VIP Levels in Serum

MTL, Gas, ET, SS, SP and VIP levels in the serum were determined by an enzyme-linked immunosorbent assay (ELISA) instrument according to the manufacturer’s instructions (Microplate Spectrophotometer Multiskan Go, Thermo Scientific, Waltham, MA, USA). A standard curve was generated from the concentration of the standard sample and the corresponding OD_450_ values. The concentration of MTL, Gas, ET, SS, SP and VIP was calculated according to the standard curve in units of ng/L. The main experimental steps in this study were as follows: first, samples (serum, standard sample, biotin-labeled secondary antibody and ELISA reagent) were added according to the manufacturer’s instructions, maintained for 1 h at 37 °C, and then washed 5 times. Then color reagents A and B were added and incubated for 10 min at 37 °C. Finally, the termination reagent was added, OD_450_ values were read, and the concentrations of MTL, Gas, ET, SS, SP and VIP were calculated.

### 4.7. Statistical Analysis

The data are presented as mean ± SD for each group. The differences between the mean values for the groups were analyzed by one-way analysis of variance with Duncan’s multiple range test. A *p* value of less than 0.05 was considered to indicate statistical significance. All statistical analyses were performed with GraphPad Prism 5 (GraphPad Software Inc., San Diego, CA, USA) and OriginPro 8.5 (OriginLab Corporation, Northampton, MA, USA).

## 5. Conclusions

In conclusion, by measuring related constipation indicators (stool status, water content of defecation, GI transit rate and time to the first black stool defecation), it was found that *B. adolescentis* CCFM 669 and 667 could alleviate constipation. In addition, *B. adolescentis* CCFM 667 exerted an effect on slow transit constipation, whereas *B. adolescentis* CCFM 626 could not. Furthermore, *B. adolescentis* CCFM 669 and 667 increased the ratio of Firmicutes to Bacteroidetes at the phylum level, increased the abundance of *Lactobacillus* and reduced the level of *Clostridium* at the genus level, and increased the amounts of propionic acid and butyric acid in feces of constipated mice. These results revealed that *B. adolescentis* (CCFM 669, CCFM 667 and CCFM 626) exerted strain-specific effects on relieving constipation. The main causes of the strain-specific effects on constipation might be the differences in the growth rate and the adhesion properties of the different strains, which exerted different effects on gut flora, the intestinal microenvironment and the related GI peptide neurotransmitter.

## Figures and Tables

**Table 1:** Tolerance of *Bifidobacterium adolescentis* strains to simulated gastric and small intestinal juices.

Strains	Time (h)					
	1	2	3	2	4	8
	Survival Rate (%)					
	Simulated Gastric Juice			Simulated Intestinal Juice		
*B. adolescentis* CCFM 626	99.30 ± 0.2	98.36 ± 0.19	85.50 ± 0.20 ^b^	81.57 ± 0.25	79.11 ± 0.41	71.51 ± 0.23 ^a^
*B. adolescentis* CCFM 669	98.16 ± 0.27	96.26 ± 0.31	81.43 ± 0.27 ^a^	78.59 ± 0.33	74.55 ± 0.26	74.55 ± 0.22 ^b^
*B. adolescentis* CCFM 667	99.91 ± 0.07	99.34 ± 0.24	94.93 ± 0.42 ^d^	90.17 ± 0.21	81.90 ± 0.19	79.50 ± 0.30 ^c^
*L. plantarum* ST-III	92.86 ± 0.28	88.28 ± 0.21	86.11 ± 0.31 ^c^	85.64 ± 0.18	81.90 ± 0.28	80.07 ± 0.28 ^d^

^a–d^ Mean values with different letters are significantly different (*p* < 0.05) according to Duncan’s multiple range test.

**Table 2:** Adhesion of different bacteria to HT-29 cells.

Strain	Adhesion
*B. adolescentis* CCFM 667	–
*B. adolescentis* CCFM 626	–
*B. adolescentis* CCFM 669	++
*Lactobacillus plantarum* ST-III	+++

Symbols: +++, >100 bacteria/cell; ++, 50–100 bacteria/cell; +, 10–50 bacteria/cell; –, <10 bacteria/cell.

**Table 3:** Short-chain fatty acids (SCFAs) in feces.

Treatment	Time (Day)	Acetic Acid (µmol/g)	Propanoic Acid (µmol/g)	Butyric Acid (µmol/g)	Total Acid (µmol/g)
Normal	7	13.71 ± 1.33 ^b^	3.25 ± 0.14 ^d^	5.00 ± 0.11 ^e^	21.96 ± 1.57 ^c^
	14	13.84 ± 1.15 ^a^	3.38 ± 0.16 ^d^	4.69 ± 0.66 ^e^	21.91 ± 1.71 ^b^
	17	13.59 ± 0.69 ^b^	3.07 ± 0.47 ^c,d^	5.57 ± 0.47 ^f^	22.23 ± 1.09 ^b^
Control	7	13.32 ± 2.19 ^b^	2.98 ± 0.3 ^d^	4.42 ± 0.36 ^d^	20.72 ± 2.11 ^b^
	14	13.57 ± 0.43 ^a^	3.28 ± 0.3 ^d^	4.92 ± 0.23 ^f^	21.77 ± 0.80 ^b^
	17	8.90 ± 0.51 ^a^	1.45 ± 0.27 ^a^	1.67 ± 0.32 ^a^	12.02 ± 0.94 ^a^
Phenolphthalein	7	7.62 ± 1.45 ^a^	1.49 ± 0.26 ^a^	2.44 ± 0.27 ^a^	11.55 ± 1.52 ^a^
	14	13.87 ± 1.96 ^a^	1.48 ± 0.26 ^a^	2.44 ± 0.27 ^a^	17.80 ± 1.89 ^a^
	17	12.66 ± 0.87 ^b^	1.53 ± 0.29 ^a^	2.98 ± 0.05 ^b^	17.17 ± 1.12 ^a^
ST-III	7	19.63 ± 1.35 ^d,e^	3.79 ± 0.29 ^e^	4.90 ± 0.06 ^e^	28.32 ± 1.22 ^f^
	14	21.60 ± 0.62 ^b^	2.70 ± 0.24 ^c^	2.94 ± 0.34 ^b^	27.24 ± 0.65 ^c^
	17	25.28 ± 2.70 ^c^	2.56 ± 0.78 ^b,c^	3.34 ± 1.15 ^c^	31.19 ± 4.33 ^c^
High dose of 669	7	24.62 ± 0.67 ^f^	2.38 ± 0.25 ^c^	3.62 ± 0.16 ^c^	30.62 ± 0.99 ^g^
	14	35.90 ± 2.29 ^d^	3.37 ± 0.28 ^d^	4.46 ± 0.36 ^e^	43.73 ± 2.76 ^f^
	17	44.19 ± 3.37 ^e,f^	3.46 ± 0.12 ^d^	5.40 ± 0.52 ^e^	53.05 ± 3.96 ^e^
Low dose of 669	7	20.13 ± 0.72 ^e^	1.76 ± 0.11 ^a,b^	2.46 ± 0.07 ^a^	24.35 ± 0.75 ^d^
	14	27.06 ± 1.12 ^c^	2.52 ± 0.12 ^b,c^	3.40 ± 0.05 ^c^	32.98 ± 1.07 ^e^
	17	47.29 ± 0.8 ^f^	3.59 ± 0.59 ^d^	3.57 ± 0.53 ^c,d^	54.45 ± 1.91 ^e^
High dose of 626	7	16.30 ± 0.32 ^c^	2.42 ± 0.23 ^c^	4.76 ± 0.24 ^e^	23.48 ± 0.28 ^d^
	14	22.97 ± 1.03 ^b^	2.24 ± 0.26 ^b^	4.24 ± 0.35 ^e^	29.45 ± 0.69 ^d^
	17	37.64 ± 1.34 ^d^	2.02 ± 0.63 ^a,b^	7.66 ± 2.05 ^g^	47.32 ± 3.94 ^d^
Low dose of 626	7	18.54 ± 0.58 ^d^	3.28 ± 0.32 ^d^	4.31 ± 0.45 ^d^	26.13 ± 1.03 ^e^
	14	20.49 ± 0.24 ^b^	2.20 ± 0.19 ^b^	3.89 ± 0.46 ^d^	26.58 ± 0.55 ^c^
	17	28.33 ± 0.59 ^c^	2.08 ± 0.01 ^a,b^	3.46 ± 0.17 ^c^	33.87 ± 0.75 ^c^
High dose of 667	7	16.15 ± 0.35 ^c^	1.88 ± 0.07 ^b^	3.21 ± 0.22 ^b^	21.24 ± 0.36 ^c^
	14	21.71 ± 6.51 ^b^	2.48 ± 0.56 ^b,c^	4.23 ± 1.23 ^e^	28.42 ± 7.42 ^d^
	17	41.29 ± 0.91 ^e^	2.37 ± 0.16 ^b,c^	5.17 ± 0.03 ^e^	48.83 ± 0.83 ^d^
Low dose of 667	7	20.45 ± 0.12 ^e^	1.88 ± 0.11 ^b^	3.63 ± 0.25 ^c^	25.96 ± 0.38 ^e^
	14	28.59 ± 0.30 ^c^	2.24 ± 0.19 ^b^	4.51 ± 0.40 ^e^	35.34 ± 0.86 ^e^
	17	43.54 ± 3.35 ^e^	3.19 ± 0.56 ^c,d^	5.25 ± 0.98 ^e^	51.97 ± 1.90 ^e^

Phenolphthalein: 70 mg/kg body weight; 626: *B. adolescentis* CCFM 626; 667: *B. adolescentis* CCFM 667; 669: *B. adolescentis* CCFM 669; ST-III: *L. plantarum* ST-III; high dose: 1 × 10^10^ CFU; low dose: 1 × 10^8^ CFU. ^a–g^ Mean values with different letters over bars are significantly different (*p* < 0.05) according to Duncan’s multiple range test.

**Table 4:** Effect size of the changes in community structure.

Groups	*p* < 0.05	
	Normal	Control
Normal	-	0.8176
Control	0.8176	-
Phenolphthalein	0.3694	0.2912
ST-III	0.4562	0.2821
High 669	0.0174	0.0074
Low 669	0.0711	0.017
High 667	0.0069	0.0032
Low 667	0.0398	0.0037
High 626	0.1436	0.0687
Low 626	0.8034	0.4104

Phenolphthalein: 70 mg/kg body weight; ST-III: *L. plantarum* ST-III; high dose: 1 × 10^10^ CFU; low dose: 1 × 10^8^ CFU.

**Table 5:** Effects of *B. adolescentis* on serum parameters in a mouse model of loperamide-induced constipation.

Groups	Test Index (ng/L)					
	MTL	Gas	SP	SS	ET-1	VIP
Normal	176.3 ± 7.25 ^c^	206.44 ± 0.18 ^d^	58.22 ± 3.4 ^c^	61.41 ± 1.5 ^a,b,c^	58.48 ± 5.99 ^b^	42.02 ± 7.14 ^a^
Control	124.15 ± 16.6 ^a^	114.37 ± 2.39 ^b^	49.2 ± 7.44 ^a,b^	96.16 ± 5.68 ^d^	105.95 ± 1.95 ^d^	67.73 ± 5.37 ^d^
Phenolphthalein	144.76 ± 4.04 ^b^	137.35 ± 12 ^b,c^	49.9 ± 5.89 ^a,b^	53.98 ± 1.08 ^a^	60.47 ± 11.69 ^b^	50.3 ± 4.36 ^c^
High dose of ST-III	174.44 ± 7.89 ^c^	126.8 ± 1.5 ^b^	54.78 ± 4.16 ^c^	64.16 ± 2.41 ^b,c^	75.94 ± 3.48 ^c^	49.05 ± 3.36 ^b,c^
Low dose of ST-III	155.35 ± 7.89 ^c^	144.76 ± 3.79 ^c^	70.04 ± 4.16 ^d^	64.16 ± 2.41 ^b,c^	75.94 ± 3.48 ^c^	70.10 ± 3.63 ^e^
High dose of 669	174.06 ± 21.68 ^c^	144.01 ± 1.3 ^c^	64.6 ± 1.51 ^c,d^	58.96 ± 3.49 ^a,b,c^	60.87 ± 3.27 ^b^	45.85 ± 5.53 ^a,b^
Low dose of 669	137.11 ± 3.09 ^a,b^	121.41 ± 8.3 ^b^	64.6 ± 1.51 ^c,d^	65.99 ± 4.38 ^c^	63.45 ± 4.88 ^b^	66.78 ± 4.34 ^d,e^
High dose of 626	143.40 ± 5.19 ^b^	132.21 ± 3.54 ^b,c^	50.86 ± 3.48 ^a,b^	97.23 ± 3.49 ^d^	91.66 ± 6.48 ^d^	61.92 ± 3.13 ^d^
Low dose of 626	138.72 ± 3.02 ^a,b^	65.66 ± 6.98 ^a^	46.99 ± 3.48 ^a^	97.33 ± 4.38 ^d^	93.68 ± 5.59 ^d^	61.92 ± 3.13 ^d^
High dose of 667	199.83 ± 10.59 ^d^	255.77 ± 27.5 ^e^	70.04 ± 1.08 ^d^	56.29 ± 0.54 ^a,b^	53.36 ± 2.13 ^a^	44.93 ± 3.57 ^a,b,c^
Low dose of 667	140.20 ± 1.93 ^b^	132.21 ± 3.54 ^b,c^	50.86 ± 1.08 ^a,b^	65.98 ± 0.54 ^c^	63.45 ± 4.88 ^b^	44.93 ± 3.57 ^a,b,c^

Phenolphthalein: 10 mg/kg body weight; 626: *B. adolescentis* CCFM 626; 667: *B. adolescentis* CCFM 667; 669: *B. adolescentis* CCFM 669; ST-III: *L. plantarum* ST-III; high dose: 1 × 10^10^ CFU; low dose: 1 × 10^8^ CFU. ^a–e^ Mean values with different letters over bars are significantly different (*p* < 0.05) according to Duncan’s multiple range test.

**Table 6:** Specifics of the animal experiment.

Group	Treatment	
	1–14 Days	15–17 Days
Normal	Normal saline solution	Normal saline solution
Control	Normal saline solution	loperamide
Phenolphthalein control	phenolphthalein	loperamide
High dose of ST-III	ST-III	loperamide
Low dose of ST-III	ST-III	loperamide
High dose of 626	626	loperamide
Low dose of 626	626	loperamide
High dose of 667	667	loperamide
Low dose of 667	667	loperamide
High dose of 669	669	loperamide
Low dose of 669	669	loperamide

Phenolphthalein: 70 mg/kg body weight; 626: *B. adolescentis* CCFM 626; 667: *B. adolescentis* CCFM 667; 669: *B. adolescentis* CCFM 669; ST-III: *L. plantarum* ST-III; high dose: 1 × 10^10^ CFU; low dose: 1 × 10^8^ CFU.
